# Global connectivity patterns of the notoriously invasive mussel, *Mytilus galloprovincialis* Lmk using archived CO1 sequence data

**DOI:** 10.1186/s13104-018-3328-3

**Published:** 2018-04-03

**Authors:** Thomas Pickett, Andrew A. David

**Affiliations:** Department of Biology, Clarkson University, Potsdam, NY 13699 USA

**Keywords:** Invasions, Population, Dispersal, Haplotype, Mytilidae

## Abstract

**Objective:**

The invasive mussel, *Mytilus galloprovincialis* has established invasive populations across the globe and in some regions, have completely displaced native mussels through competitive exclusion. The objective of this study was to elucidate global connectivity patterns of *M. galloprovincialis* strictly using archived cytochrome c oxidase 1 sequence data obtained from public databases. Through exhaustive mining and the development of a systematic workflow, we compiled the most comprehensive global CO1 dataset for *M. galloprovincialis* thus far, consisting of 209 sequences representing 14 populations. Haplotype networks were constructed and genetic differentiation was assessed using pairwise analysis of molecular variance.

**Results:**

There was significant genetic structuring across populations with significant geographic patterning of haplotypes. In particular, South Korea, South China, Turkey and Australasia appear to be the most genetically isolated populations. However, we were unable to recover a northern and southern hemisphere grouping for *M. galloprovincialis* as was found in previous studies. These results suggest a complex dispersal pattern for *M. galloprovincialis* driven by several contributors including both natural and anthropogenic dispersal mechanisms along with the possibility of potential hybridization and ancient vicariance events.

**Electronic supplementary material:**

The online version of this article (10.1186/s13104-018-3328-3) contains supplementary material, which is available to authorized users.

## Introduction

Quantifying dispersal in marine environments has been a long standing challenge due to the difficulty in tracking large numbers of microscopic larvae within oceanic basins [1]. As a consequence, indirect methods have been developed, the most common of which is population genetics. In marine invasion ecology, population genetics is often employed to track the dispersal of invasive species but the dynamic nature of marine invasions caused by changes in vector strength, transient dispersal barriers and stochastic factors, poses a challenge [2]. One potential alternative could be the use of archived sequence data which possess a temporal element. As sequencing costs continues to decline, public data banks that archive sequences are growing at an exponential rate [3]. These data banks play a central role in the life sciences because they allow for the reproducibility of published research, which recently, has been a contentious issue in the life sciences [4]. In invasion genetics and indeed, population genetics as a whole, such resources remain surprisingly underutilized [3], despite the fact that they possess a wealth of spatio-temporal sequence data generated from a variety of projects [5, 6]. In this study, we attempted to elucidate global connectivity patterns of the invasive Mediterranean mussel, *Mytilus galloprovincialis* Lamarck, 1819 by repurposing archived cytochrome c oxidase 1 (CO1) sequence data from public databanks.

*Mytilus galloprovincialis* is a relatively small marine bivalve (5–8 cm) that is native to the Mediterranean but has aggressively extended its range to the Americas, Asia, southern Africa and Australasia [7–9]. The primary vector responsible for the spread of this species includes shipping, more specifically the transportation of planktonic larvae in ballast water of commercial ships and attachment of byssal threads to ship hulls [10]. In addition, the ease of culturing *M. galloprovincialis* along with its palatability have resulted in the transplantation of stock populations for aquaculture purposes in different regions of the world [10]. The success of the species in its introduced range is due to inherent biological characteristics that make it an aggressive invader, including high fecundity and recruitment rates [11], broad thermal tolerance [12] and resistance to desiccation and parasites [13]. The objective of this study was to assess levels of genetic differentiation of *M. galloprovincialis* across multiple global localities. We hypothesized that *M. galloprovincialis* would exhibit marked genetic differentiation between northern and southern hemisphere mussels but overall very low levels of genetic differentiation within hemispheres due to repeated introductions.

## Main text

### Materials and methods

#### Data mining and alignment

**Fig. 1:**
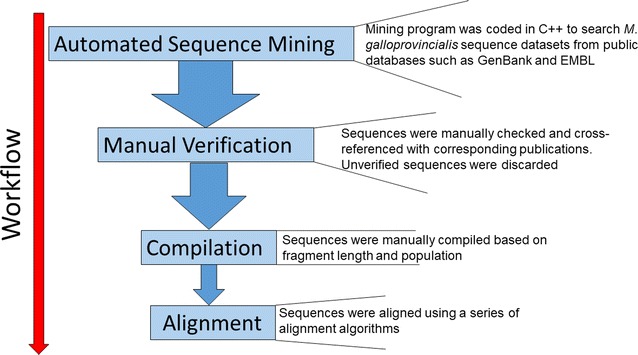
Workflow for CO1 sequence acquisition of *Mytilus galloprovincialis* from data mining to sequence alignment

**Fig. 2:**
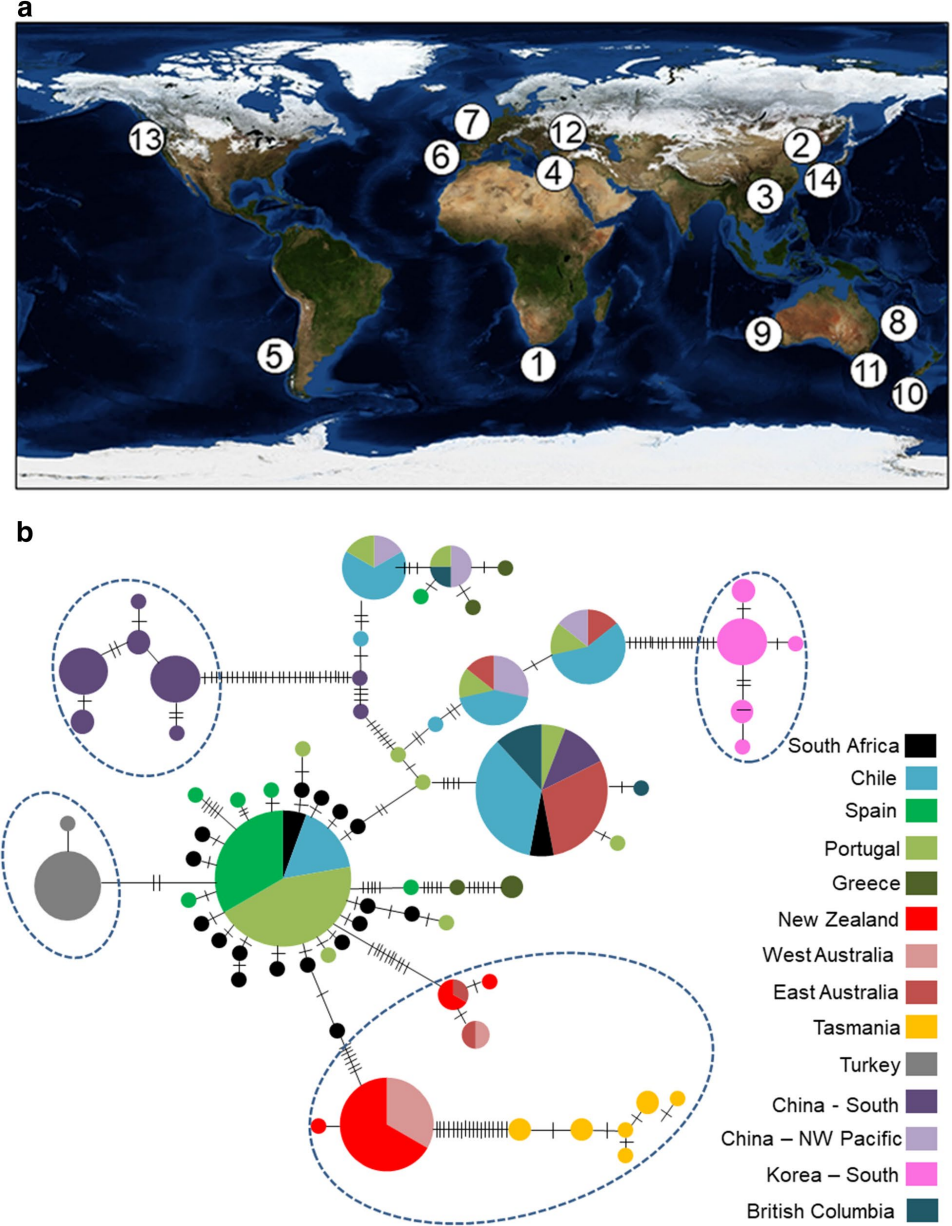
**a** Distribution map of *Mytilus galloprovincialis* CO1 sequences: 1—South Africa, 2—Northwest Pacific (NP) China, 3—South China, 4—Greece, 5—Chile, 6—Portugal, 7—Spain, 8—Australia East, 9—Australia West, 10—New Zealand (Auckland Islands), 11—Tasmania, 12—Turkey, 13—British Columbia (Vancouver Island), 14—Korea (South). Map Credit: Reto Stöckli, NASA Earth Observatory. **b** Haplotype network for *Mytilus galloprovincialis* based on mtDNA—CO1 sequence data. Size of circles is representative of individuals with that haplotype. The smallest circles represent a haplotype frequency of one. Each connecting line between haplotypes represents one mutational step and perpendicular lines represent an additional mutational change. Dashed circles indicate distinct haplogroups

A workflow for repurposing repository sequence data was developed (Fig. 1). A mining program was first coded in C++ to search several DNA databases for *M. galloprovincialis* DNA sequences. These databases included GenBank, the European Nucleotide Archive, DNA Database of Japan and the Barcode of Life Database (BoLD). An automated search was preferentially chosen over a manual search due to the speed of sequence acquisition, and its highly discriminative nature (a specific code is unlikely to pull duplicates, or ambiguous sequences). The mitochondrial DNA marker, cytochrome c oxidase 1 (CO1) was chosen due to its overrepresentation in population genetic studies for this species relative to other markers. The following qualifiers were incorporated into the coding script: ‘Mytilus’, ‘galloprovincialis’ ‘mitochondrial’, ‘CO1’. After scanning 2480 mitochondrial gene sequences, 322 CO1 fragments were recovered (date of original search: April 2016). For verification, each database entry was manually checked and discarded if: (i) the sequence could not be linked to published research (peer reviewed articles, technical papers or conference abstracts) and or (ii) the sequence could not be traced to a specific geographic locality. In a few cases the mining program recovered ‘CO1-like sequences’ which were discarded. To avoid compiling duplicated sequences, database entries were cross referenced and any duplications were also discarded. To confirm collection dates, sequences were cross-referenced to their corresponding publications and in cases where no collection date was specified, authors were contacted directly for confirmation. Based on these aforementioned filters, a finally tally of 209 sequences representing 14 distinct populations were tagged as ‘useable’ for this study and they were all accessible from the GenBank database (Additional file 1: Table S1; Fig. 2a). A series of alignment algorithms (CLUSTALW, MUSCLE and MAFFT) were tested on the compiled dataset set in Geneious ver 10.1.3 [14] and edited in BioEdit ver. 5 [15]. The MAFFT algorithm provided the highest quality dataset as measured by bp length. It was also chosen because to its incorporation of iterative refinement steps that corrects for accidental misalignments [16].

#### Genetic differentiation analyses

To determine evolutionary relationships among haplotypes, a statistical parsimony network was constructed using TCS ver.1.2.1 [17], with the fixed connection limit set to 95%. Genetic differentiation across populations was calculated via pairwise ɸ_ST_ comparisons that were carried out in Arlequin ver. 3.5. [18]. To determine the extent of differentiation between northern and southern hemisphere populations, populations representing both regions of the world were clustered into batches: (i) North: Spain, Portugal, British Columbia, Turkey, China (South and Northwestern Pacific—NP), Korea, Portugal and (ii) South: South Africa, Chile, Australia (West and East), Tasmania and New Zealand. A hierarchical analysis of molecular variance (AMOVA) along with ɸ_ST_ calculations were carried out on both clusters and among sites to estimate the extent of genetic differentiation.

### Results

A 360-bp fragment with 157 variable sites was obtained for the CO1 marker. A total of 67 haplotypes was recovered of which 47 were unique and 38% of these unique haplotypes originated from the South African population (Fig. 2b). There were four distinct haplogroups that, with the exception of Turkish individuals, were separated by at least 20 mutational steps. These haplogroups included Australasia, consisting of some Australian individuals and the entire Tasmanian and New Zealand populations, Turkey, Korea and South China. While the parsimony network showed generally strong geographic patterning of haplotypes, some haplotypes were shared by individuals from six geographically distinct populations. There was also a close relationship between South African and Chilean haplotypes with North Atlantic and Mediterranean haplotypes as indicated by the high frequency of haplotype sharing and the small number of mutational steps separating them.

The ɸ_ST_ value between northern and southern hemisphere localities showed low but significant genetic partitioning (ɸ_ST_ = 0.11, P < 0.01) with 88% of the genetic variation nested within individual hemispheres. Pairwise comparisons showed generally high ɸ_ST_ values indicating strong genetic differentiation across all localities. Tasmania, South China and South Korea were the most genetically isolated populations, with all three generating significant ɸ_ST_ values of 0.80–0.93 when compared with other populations (Table 1). The least genetically differentiated populations were between the North Atlantic and Mediterranean populations (ɸ_ST_ = 0.07–0.26) and the Chilean and North Atlantic/Mediterranean populations (ɸ_ST_ = 0.07–0.13). There was also low and non-significant genetic differentiation between British Columbia and Chile, China (NP), Portugal and western Australia (0.07–0.18).

**Table 1:** Pairwise ɸ_ST_ values for *M. galloprovincialis* using the CO1 gene

Locality	South Africa	China (South)	China (NP)	Chile	Portugal	Greece	Spain	Australia East	Australia West	Tasmania	New Zealand	Turkey	South Korea	British Columbia
South Africa	–													
China (South)	*0.90*	–												
China (NP)	*0.60*	*0.90*	–											
Chile	*0.21*	*0.80*	0.07 (0.34)	–										
Portugal	*0.35*	*0.90*	*0.21*	*0.13*	–									
Greece	*0.52*	*0.92*	*0.27*	0.07 (0.09)	*0.15*	–								
Spain	*0.35*	*0.93*	*0.44*	*0.14*	0.08 (0.09)	*0.26*	–							
Australia East	*0.68*	*0.92*	*0.15*	0.08 (0.11)	*0.15*	*0.27*	*0.37*	–						
Australia West	*0.86*	*0.93*	*0.63*	*0.41*	0.65 (0.12)	*0.63*	*0.66*	*0.61*	–					
Tasmania	*0.86*	*0.92*	*0.74*	*0.24*	*0.73*	*0.80*	*0.81*	*0.70*	*0.56*	–				
New Zealand	*0.82*	*0.92*	*0.71*	*0.24*	*0.69*	*0.75*	*0.76*	*0.65*	*0.63*	*0.76*	–			
Turkey	*0.69*	*0.93*	*0.68*	*0.20*	*0.43*	*0.68*	*0.51*	*0.63*	*0.70*	*0.95*	*0.88*	–		
South Korea	*0.92*	*0.91*	*0.88*	*0.52*	*0.90*	*0.91*	*0.93*	*0.90*	*0.83*	*0.92*	*0.90*	*0.95*	–	
BC	*0.63*	*0.94*	0.09 (0.32)	0.10 (0.24)	0.18 (0.07)	*0.30*	*0.45*	0.07 (0.51)	*0.61*	*0.83*	*0.77*	*0.85*	*0.92*	–

NP, North-West Pacific, BC, British Columbia (Vancouver Island)

P-values are shown in brackets and values in italics are significant (P < 0.05)

### Discussion

Previous phylogenetic studies using RFLPS and 16S rRNA sequences recovered two distinct northern and southern hemisphere clades of *M. galloprovincialis* [19–21]. In contrast, the CO1 gene in this study failed to recover these such grouping and in fact, hierarchical AMOVA results found that most of the genetic variation (~ 88%) was found within the southern and northern populations rather than between them. This issue is of particular importance because a distinct northern and southern clade of *M. galloprovincialis* is used as evidence for the support of the ‘northern migration’ hypothesis which states that *M. galloprovincialis* diverged from an ancestral *M. edulis* in the Mediterranean Sea more than 1.5 mya and then migrated to the southern hemisphere during the Pleistocene via the Atlantic Ocean. The lack of distinct northern and southern hemisphere groupings could be due to cryptic dispersal where frequent anthropogenic transport could dilute phylogeographic signal and consequently decrease the value of standard population genetic parameters (e.g. F_ST_) [22].

While our study did not recover a northern and southern group, it did recover two Australasian haplogroups consisting of some individuals from east Australia and the entire western Australian, Tasmanian and New Zealand population. Approximately 99% of *Mytilus* mussels sampled in Australia are *M. galloprovincialis* [23]. In particular, Tasmanian individuals did not share haplotypes with any other population and were even genetically isolated from nearby Australian and New Zealand individuals. These results are congruent with a previous nuclear hybridization study which showed that Tasmanian *M. galloprovincialis* is endemic and a secondary contact with *M. edulis* either before or after the founding population became established, could have resulted in the genetic isolation observed in the present study [24].

Both South Korean and South Chinese *M. galloprovincialis* populations also showed genetic isolation based on haplotype networks and pairwise AMOVA results. More surprising, however was that the Korean population was strongly differentiated from both the NP Chinese and South Chinese populations (ɸ_ST_ = 0.88 and 0.91 respectively—P < 0.05) despite the fact that all three localities are located less than 2000 km away from each other in the Yellow Sea with ocean current direction conducive to fine scale connectivity. The high genetic structure observed in this region could be due to hybridization. In all three Asian localities, *M. galloprovincialis*’ range overlaps with that of *M. edulis* and *M. trossulus*. [25]. Hybridization and mtDNA introgression among these species are common in regions where they occur together [7]. When interspecific hybridization occurs, especially during invasion events, there is an elevated response to selection pressures, resulting in rapid rates of adaptation and as a consequence, the development of barriers to mtDNA exchange [26]. It is therefore possible that our sequence alignment may have included hybrid lineages that were submitted under the name of *M. galloprovincialis* though further genetic analyses will be needed to definitively detect the presence of hybridization. Alternatively, past geological and climatic changes in this coastal system could have resulted in ancient vicariance events [27], leading to the observed genetic structuring of the East Asian populations.

There were also frequent instances of haplotype sharing along between NP Chinese populations, North Atlantic, Mediterranean and Pacific populations of Chile and British Columbia, which is congruent with past population studies of *M. galloprovincialis* using nuclear markers [28–30]. In addition, we found that the North Atlantic and Mediterranean population shared haplotypes with the NP Chinese, Chilean and British Columbia populations. Since it is unlikely that such close kinship is due to dispersal across the Pacific Ocean, we hypothesize that multiple introductory events could be connecting these populations. Previous surveys and studies have shown that the Pacific, especially the northwestern Pacific has been a viable corridor for non-indigenous species for decades [31]. Transoceanic shipping across this corridor could therefore be responsible for the movement of *M. galloprovincialis* from the Asian Pacific region to British Columbia.

The negative impacts of *M. galloprovincialis* has been studied extensively on the southern African coast where the mussel had rapidly colonized the western coast of the country upon in its introduction in the 1970s and has since displaced the native mussel *Aulacomya ater* in this region [8]. Both haplotype networks and AMOVA results agrees with previous studies that suggest a Mediterranean origin for South African *M. galloprovincialis* [32].

In conclusion, our results are indicative of a complex dispersal pattern for *M. galloprovincialis* and it likely involves a combination of natural and anthropogenic dispersal coupled with local adaptation and hybridization events. Most importantly, these findings were based on archived genetic data drawn from disparate studies. This study therefore shows that DNA sequence repositories possess valuable genetic data, from which informative results can be gleaned from post hoc analyses.

### Limitations

Many researchers are very wary of using public databases to test new hypotheses especially in large scale population genetic studies. A significant issue is the taxonomic reliability of submitted sequences, especially with regards to closely related species like the *Mytilus* spp. complex of which the study species belongs to. However, we believe our workflow for repurposing sequence data was adequate in filtering bona fide *M. galloprovincialis* CO1 sequences. However, we did encounter some problems associated with sequence acquisition itself. For some of the DNA barcoding and phylogenetic studies, sampling localities were not included either as a source modifier in GenBank or in the published study, and in such cases authors had to be contacted directly for this information. In addition, GPS co-ordinates were also missing so we were unable to carry out some crucial tests commonly used in connectivity studies including isolation by distance (IBD) calculations and SAMOVA (spatial analysis of molecular variance).

## Additional file

**Additional file 1: Table S1.** GenBank accession data. List of GenBank accession numbers for all CO1 sequences used in the present study. In this file, sequences are separated by population and both sample size and the original purpose of the sequences are provided.

## Abbreviations

CO1: cytochrome c oxidase 1
BoLD: Barcode of Life Database
AMOVA: analysis of molecular variance
RFLPS: restriction fragment length polymorphism
rRNA: ribosomal RNA
NP: North Pacific
IBD: isolation by distance
SAMOVA: spatial analysis of molecular variance
