# Supplementary material for: Global connectivity patterns of the notoriously invasive mussel, Mytilus galloprovincialis Lmk using archived CO1 sequence data
Source: BMC Res Notes. 2018 Apr 3;11:231. doi: 10.1186/s13104-018-3328-3 (PMC5883410; doi:10.1186/s13104-018-3328-3)
Supplement: Supplementary file 1 — Additional file 1: Table S1. GenBank accession data. List of GenBank accession numbers for all CO1 sequences used in the present study. In this file, sequences are separated by population and both sample size and the original purpose of the sequences are provided. [file 13104_2018_3328_MOESM1_ESM.docx]

Table S1. Verified CO1 gene sequences of *Mytilus galloprovincialis* mined from GenBank

| **Population** | **Sample size** | **GenBank Accession numbers** | **Original purpose of dataset** |
| --- | --- | --- | --- |
|  |  |  |  |
| South Africa (1) | 20 | DQ351478 – DQ351497 | phylogeography |
| China – Northwest (2) | 11 | KU201434 – K201444 | phylogeography |
| China – South (3) | 12 | GU566680 – 566691 | Genetic diversity studies |
| Greece (4) | 6 | DQ469132, DQ445475. DQ445472, DQ445469, DQ445466, DQ403169 | fertility studies |
| Chile (5) | 26 | KP052901 – KP052927 | barcoding, biodiversity study |
| Portugal (6) | 19 | KC819224 – KC819242 | invasion genetics, population genetics |
| Spain (7) | 13 | KU697775 – KU697787 | invasion genetics, biodiversity study |
| Australia – East (8) | 10 | DQ864416 – DQ864425 | conservation genetics, barcoding |
| Australia – West (9) | 37 | KF705213 – KF705244 | aquaculture, barcoding |
| New Zealand –Auckland Islands (10) | 12 | DQ864378 – DQ864387 | conservation genetics, barcoding |
| Tasmania (11) | 10 | DQ864388 – DQ864396 | conservation genetics, barcoding |
| Turkey (12) | 16 | KC789258 – KC789273, KC311404 | barcoding, biodiversity studies |
| British Columbia – Vancouver Island (13) | 8 | KF931759 – KF931762 | aquaculture, hybridization studies |
| Korea – South (14) | 8 | HM180705 – HM 180712 | barcoding study |
